# Dietary quality score is positively associated with serum adiponectin level in Indonesian preschool-age children living in the urban area of Jakarta

**DOI:** 10.1371/journal.pone.0246234

**Published:** 2021-02-04

**Authors:** Anastasia Hayuningtyas, Yayang Aditia Dewi, Lestari Octavia, Aman Pulungan, Rina Agustina

**Affiliations:** 1 Department of Nutrition, Faculty of Medicine, Universitas Indonesia—Dr. Cipto Mangunkusumo General Hospital, Jakarta, Indonesia; 2 Department of Child Health, Faculty of Medicine, Universitas Indonesia—Dr. Cipto Mangunkusumo General Hospital, Jakarta, Indonesia; 3 Human Nutrition Research Centre, Indonesia Medical Education and Research Institute Indonesia, Faculty of Medicine, Universitas Indonesia, Jakarta, Indonesia; Copenhagen University Hospital Holbæk, DENMARK

## Abstract

An unhealthy diet during childhood directly impacts the risk of developing noncommunicable diseases (NCDs) later on in life. However, well-documented information on this issue is lacking. We investigated the dietary quality of young Indonesian children and assessed the relationship to serum adiponectin levels as an early marker of NCDs. Eighty-five (44 girls and 41 boys) Indonesian preschool-age children in East Jakarta were included in this study. Dietary intake data were gathered by collecting repeated 24-hour recalls for one weekday and one day during the weekend, which were then further converted into participants’ Healthy Eating Index (HEI) 2015 scores. Meanwhile, an enzyme-linked immunosorbent assay was performed to determine the serum adiponectin level. A multiple regression analysis was performed to assess the association between the HEI 2015 score and serum adiponectin, adjusting for potential confounders. The mean HEI 2015 score was 33.2 ± 8.3 points, which was far below the recommended score of ≥ 80 points, while the mean serum adiponectin was 10.3 ± 4.1 μg/mL. Multiple linear regression testing showed that a one-point increase in the HEI 2015 score was significantly associated with an increase in the serum adiponectin level by 0.115 μg/mL after adjusting for exclusive breastfeeding history (β = 0.115; 95% CI = 0.010–0.221; p = 0.032). In conclusion, better adherence of young children to a healthy diet has a positive association with their adiponectin level. This result suggests that strengthening children’s dietary quality from an early age by involving all parties in the children’s environment (e.g., parents, teachers at school, policymakers) may help to reduce the risk of NCDs later on in childhood and during adult life.

## Introduction

In 2015, world leaders endorsed the Sustainable Development Goal as a global development agreement aimed at reducing poverty and inequality and protecting the environment. Among the 17 key goals that compose this initiative, reducing premature death rates due to noncommunicable diseases (NCDs) by 30% by 2030 is one of the health sector targets [1]. In 2018, the Indonesian national basic health survey highlighted a trend of increasing NCDs, which include diabetes mellitus, hypertension, and obesity, as compared with the findings of the previous survey performed in 2013 [2, 3]. This trend was also reported in one study of obese adolescents in Jakarta in 2013, where the prevalence of insulin resistance and hypertension was high [4]. Although NCDs primarily affect adults, the risk levels for many NCDs become established early in life [5], begging further assessments of childhood dietary behaviors.

Adiponectin, a hormone produced by adipose tissue, has been widely studied regarding its role in obesity, diabetes, inflammation, atherosclerosis, and cardiovascular disease [6]. It plays a role in suppressing hepatic gluconeogenesis and increasing the oxidation of fatty acids in the skeletal muscle. It also protects cells from apoptosis and reduces cell inflammation [6]. Found in both the adult population [7, 8] and the pediatric population [9], low adiponectin levels can be detected in similar conditions such as obesity, high blood pressure [10, 11], and insulin-resistant state [12]. Previous studies have also indicated that low adiponectin levels during childhood can predict an increased intima-media thickness in adulthood [13, 14]. These findings suggest the benefits of monitoring adiponectin levels in childhood.

Lifestyle changes from childhood may aid in preventing NCDs in adulthood [9]. Diet is one of many lifestyle factors that can modulate adiponectin levels in the body. Moreover, earlier exposure to healthy eating patterns can increase children’s likelihood of adhering to healthier eating habits when they grow up [15]. Previous studies in adolescent and adult populations have reported a positive relationship exists between healthy diet patterns such as the Mediterranean diet, low glycemic index diets, high-fiber diets, and adiponectin concentrations in the blood [16–18]. However, evidence regarding this association in pediatric populations is still lacking. Moreover, many studies in this population focused on the association with caloric restriction or macronutrient intake rather than looking at the whole diet [19–21]. Thus, the results are harder to conclude into an applicable dietary recommendation.

One indicator for measuring the overall diet quality is the Healthy Eating Index (HEI) [22, 23], which is suitable to assess young children’s dietary quality because its components are evaluated on a density basis (amounts per 1,000 calories). Generally, dietary recommendations differ based on age, sex, and activity level. However, most are surprisingly comparable when looked at on a 1,000 calorie basis [24].

The HEI has been deployed to date in many countries such as Indonesia [25], Thailand [26], China [27], and other low- and middle-income countries (LMICs) [28], albeit largely in the adult population. Meanwhile, although similar studies in preschool-age children have been performed in developed countries such as the United States of America [29] and Portugal [30], pediatric studies of HEI in developing countries are still limited [31]. Also, studies that have examined the relationship between dietary quality and metabolic disease markers in young Indonesian children are rare. The purpose of this study was therefore to identify Indonesian children’s overall diet quality using the HEI and to assess its association with serum adiponectin level as an early marker of NCDs.

## Materials and methods

### Study design and site

This cross-sectional study was performed between September 2018 and May 2019 as part of the existing East Jakarta Cohort Study (EJCS) initiated in 2014 and revisited in 2018 in 10 subdistricts in East Jakarta, Indonesia [32]. The Ethics Committee of the Faculty of Medicine, Universitas Indonesia, approved this study (no: 1114/UN2.F1/ETIK/IX/2018). The study was registered at ClinicalTrials.gov under the identifier NCT04096521.

### Study participants and sampling procedure

The study participants were children whose mothers were listed in the EJCS study. Eligible children included those who were healthy and of preschool age (3–5 years old); residing in East Jakarta during the period when this study took place; without a history of hormonal diseases; and without taking long-term drugs such as steroids, antipsychotic drugs, or metabolic disease medications. Before the study began, parents or primary caregivers who agreed to their children’s inclusion in this study voluntarily signed written informed consent statements.

A previous study by Kashino et al. found a correlation between Japanese dietary patterns and adiponectin level (r = 0.3; p < 0.05) [33]. An a priori analysis was conducted to calculate the minimum sample size needed to find the correlation between the HEI 2015 score and serum adiponectin. Using a two-tailed test with a correlation of 0.30 and an alpha value of 0.05 [34], at least 84 participants were deemed necessary to achieve a power of 0.80.

The initial pregnant women that were sourced from the 2014 EJCS study totaled 282 people [32], while, in the 2018 EJCS follow-up study, 127 children were followed up with and assessed. Two of these children were later excluded because their age was younger than three years old. Of the remaining 125 children, the caregivers of 40 of them declined the blood examination. In the end, the total sample size of this study was 85 children. There were no significant differences in the baseline characteristics between the initial sample (n = 127) and the region population [35], or between study responders (n = 85) and non-responders (n = 40). These comparisons are quantified in greater detail in the S1 Table.

### Data collection

We developed a questionnaire consisting of several parts, including general sociodemographic characteristics, dietary intake, and anthropometric data (i.e., history of birth weight and length, current examination data of body weight, body height, and waist circumference). One month before the study began, we conducted a preliminary survey on a population of 30 preschool-age children with similar characteristics, separate from the research participants. Cronbach’s alpha analysis (α = 0.78) has been carried out to ensure the questionnaire’s reliability. Trained enumerators executed the interview and anthropometry examination. Nonfasting blood collection was completed in the morning before noon from the children by professional phlebotomists to measure the adiponectin level [36].

#### Sociodemographic information

A structured questionnaire was used to acquire data regarding the child’s age, sex, exclusive breastfeeding history, physical activities (i.e., sedentary activities, moderate and vigorous activities, and sleep duration), parents’ NCD history, parents’ education level, and family income level. The exclusive breastfeeding history was determined by how long the children had consumed only breastmilk from birth. Data on daily sedentary and moderate to vigorous activities were collected in minutes, while daily sleep-duration data were collected in hours.

#### Dietary assessment

Food intake data were obtained through interviews using a 24-hour food recall questionnaire [37]. We used a food consumption photograph book with images of local foods [38] to help the field enumerators and assist the children’s caregiver in determining types and portion sizes of food. During the interview, we asked the caregiver about the type and amount of food consumed by their child in the last 24 hours. This interview was conducted twice, once on a weekday and once during the weekend [39]. Then, we converted the food intake data into grams by using a list of food ingredients analysis and a list of food ingredients exchangers. Food intake data were analyzed using the Nutrisurvey 2007 software program.

The intake data were further converted into the HEI 2015 score [23], which consists of 13 components. These components are divided into two groups (adequacy and moderation) [24]. The adequacy group is that from which intake is encouraged; it consists of total fruit, whole fruit, total vegetable, greens and beans, whole grains, dairy, total protein foods, seafood and plant protein, and fatty acids components [24]. On the other hand, the moderation group includes food of which intake must be limited—namely, refined grains, sodium, added sugar, and saturated fat components [22, 23].

In the adequacy component, a higher score indicates greater consumption. In contrast, in the moderation component, a higher score indicates lower consumption. The total HEI 2015 score ranges from zero to 100 points; a score above 80 points indicates a good diet quality, a score between 51 and 80 points indicates an adequate diet quality (needs improvement), and a score below 51 points indicates a poor diet quality (significant revision required) [24].

#### Anthropometric measurement

Study participants’ birth weight and birth length data were collected during the interview. Low birth weight history was defined as when the birth weight was 2,500 g or less, and low birth length history was defined as when the birth length was less than 48 cm.

Each anthropometric measurement was taken twice and the two values were averaged. Body weight was measured in kilograms using a calibrated SECA^®^ digital scale (Medisave UK Ltd., UK) with a precision of 0.1 kg. Body height was measured using calibrated ShorrBoard^®^ (Weigh and Measure LLC, USA) with an accuracy of 0.1 cm. Waist circumference was measured with a SECA^®^ measuring tape (Medisave UK Ltd., UK) at the midpoint between the adjacent last ribs and the iliac crest’s peak at the end of normal expiration with the participant standing upright, feet together, arms at the sides, without the measured part covered by clothes, with a precision of 0.1 cm [40]. Participants were defined as having abdominal obesity if their waist circumference according to age and sex was in the 80th percentile or above [41].

This study adopted the body mass index (BMI) for age and sex percentile growth chart from the Centers for Disease Control and Prevention [42, 43] as this chart is sensitive for detecting adiposity in young children and can be utilized continuously from two until 20 years of age, making growth monitoring throughout the life cycle more manageable [43–45]. Body Mass Index was calculated by dividing the body weight (in kilogram) by the square of the body height (in meters squared); then, the result was compared against the BMI for age and sex percentile growth chart [42, 43]. The BMI for age and sex percentiles were classified into four groups: underweight (<5^th^ percentile), normal weight (5^th^ - 85^th^ percentile), at risk of being overweight (≥ 85^th^ percentile), and overweight (≥ 95^th^ percentile) [46]. In the current study, we combined participants at risk of being overweight and those who were overweight into one group.

#### Laboratory examination

Blood samples were drawn by vein puncture. Serum samples were separated from whole blood by centrifugation and immediately stored at –20°C until assay. Serum adiponectin (μg/mL) concentrations were established using the enzyme-linked immunosorbent assay (ELISA) technique [47] in a standardized laboratory.

As reference data on serum adiponectin values for the pediatric population in Indonesia are not available yet, we used the cutoff values from the Identification and prevention of dietary-and-lifestyle-induced health effects in children and infants (IDEFICS) cohort study to ensure that our participants’ adiponectin values were within the typical range [48]. The IDEFICS cohort study also used the ELISA method to measure adiponectin levels and determined adiponectin reference values specifically based on age and sex: for children aged three to four years, adiponectin’s reference value in girls are between 6.14 and 22.7 μg/mL, while those in boys are between 5.12 and 24.9 μg/mL. Additionally, for children aged four to five years, the values for girls are between 5.59 and 22.49 μg/mL and, for boys, they are between 4.75 and 23.11 μg/mL [48].

### Statistical analysis

The Kolmogorov-Smirnoff test was used to determine the normal distribution of continuous variables. Categorical data were presented in the form of n (%), while continuous data were presented as mean ± standard deviation values if normal distribution data or median (25^th^-75^th^ percentile) values if their distribution was not normal [49].

Bivariate analysis was performed to determine the variables that potentially become the confounding factors for serum adiponectin levels, marked with p < 0.25. A multiple linear regression test was then performed to analyze the relationship between the HEI 2015 score and serum adiponectin concentration after adjusting for potential confounders. Multiple regression coefficients (β) were described using 95% confidence interval (CI) and p-values of less than 0.05 were considered as statistically significant [49]. Statistical analyses were performed using the Statistical Package for the Social Sciences (SPSS) version 20.0 software program (IBM Corporation, USA).

## Results

### Participants’ general characteristics

A total of 85 children (44 girls and 41 boys) were included in the analyses. Their general characteristics were presented in **Table 1**.

**Table 1:** General characteristics of preschool-age children in the urban area of Jakarta and its association with serum adiponectin (n = 85).

Variables	Values^1^	Association with adiponectin	
		R-Value	P-Value*
Age (year)	3.4 (3.3–3.4)	0.057	0.604^P^
Gender			0.541^T^
Female	44 (51.8)		
Male	41 (48.2)		
Had low birth weight history^2^	4 (4.7)		0.870^T^
Had low birth length history^3^	20 (23.5)		0.677^T^
Exclusive breastfeeding history			0.093^T^
< Six months	41 (48.2)		
≥ Six months	44 (51.8)		
Light physical activity^4^			0.540^T^
Sufficient	46 (54.1)		
Low	39 (45.9)		
Moderate to vigorous activity^5^			0.266^T^
Low	10 (11.6)		
Sufficient	75 (88.2)		
Total sleep^6^			0.482^T^
Low	10 (11.8)		
Sufficient	75 (88.2)		
BMI for age and sex percentile category^7^			0.040^A^
Underweight	13 (15.3)		
Normal weight	58 (68.2)		
Risk of overweight to overweight	14 (16.5)		
Having abdominal obesity^8^	15 (17.6)		0.089^T^
Parent NCDs^9^ history			
Mother had an NCDs history	20 (23.5)		0.774^T^
Father had an NCDs history	21 (24.7)		0.906^T^
Mother education level^10^			0.830^T^
Low	26 (30.6)		
Medium to high	59 (69.4)		
Father education level^10^			0.566^T^
Low	22 (25.9)		
Medium to high	63 (74.1)		
Household income classification^11^			0.628^T^
Low	51 (60.0)		
Sufficient	34 (40.0)		

^1^ Variable presented in mean ± SD or n (%).

^2^ Low birth weight: birth weight ≤ 2500 g.

^3^ Low birth length: birth length < 48 cm.

^4^ Sufficient (≤60 min/d), Low (>60 min/d).

^5^ Low (<60 min/d), Sufficient (≥60 min/d).

^6^ Low (<10 hours/d), Sufficient (≥10 hours/d).

^7^ Body Mass Index for age and sex percentile category: [1] <25^th^ percentile = underweight, [2] 25^th^-85^th^ percentile = normal weight, [3] >85^th^ percentile = risk of overweight to overweight.

^8^ Abdominal obesity status based on waist circumference reference for age and sex: No (<80^th^ percentile), yes (≥80^th^ percentile).

^9^ Noncommunicable diseases (hypertension, diabetes mellitus, heart disease, dyslipidemia).

^10^ Low (below or equal to the 9-year compulsory education); Medium to high (over the mandatory 9-year education).

^11^ Low: below the provincial minimum wage of Jakarta 2018 (< IDR 3,648,035 or < USD 250); Sufficient: equal to or over the provincial minimum wage of Jakarta 2018 (≥ IDR 3,648,035 or ≥ USD 250).

^P^ Pearson correlation coefficient.

^T^ Independent-Samples T-Test.

^A^ Analysis of variance (ANOVA) test.

* Statistically significant if p<0.05.

The median age of the children was 3.4 years old. More than half of the children came from low-income families (60%), and most of them did not have low birth weight (95.3%) or low birth length history (76.5%). Half of the children had exclusive breastfeeding histories for equal to more than six months. Most of the subjects have sufficient physical activity levels as indicated with sedentary activities of ≤ 60 minutes/ day, moderate and vigorous activities of ≥ 60 minutes/ day, total sleep duration of ≥ 10 hours/ day. These references were based on the WHO recommendation for physical activity under five-year-old children [50]. According to the median BMI for age and sex percentile assessment, 16.5% of the children were categorized into the ‘risk of overweight to overweight’ group. Meanwhile, we found that 17.6% of the children had abdominal obesity from the waist circumference data. Most of the parents did not have NCDs history and surpassed the minimum education level obliged by the government.

### Healthy Eating Index and serum adiponectin level

The exact value of each component of HEI 2015 was outlined in **Table 2**. All subjects had a total HEI 2015 score of less than 80, with the mean of the HEI 2015 overall score was 33.2 ± 8.3, indicating poor diet quality. The mean serum adiponectin level was 10.3 ± 4.1 μg/mL.

**Table 2:** Healthy Eating Index 2015 score and its association with serum adiponectin (n = 85).

Component	Max Score	Subjects’score^1^	Association with adiponectin	
			R-Value	P-Value
Total HEI 2015	100	33.2 ± 8.3	0.227	0.037^P^*
Adequacy components^2^				
Total fruit	5	0 (0–0.9)	0.197	0.071^P^
Whole fruit	5	0 (0–1.7)	0.181	0.098^P^
Total vegetable	5	0.7 (0.3–1.4)	-0.147	0.178^P^
Green and beans	5	0.2 (0–1.2)	0.101	0.359^P^
Whole grains	10	0 (0–0)	-0.050	0.648^P^
Dairy	10	3.3 ± 2.5	0.016	0.885^P^
Total protein foods	5	3.8 (2.2–5)	-0.087	0.427^P^
Seafood and plant protein	5	1.9 (0.2–4.7)	0.038	0.730^P^
Fatty acids	10	0 (0–0)	0.114	0.296^P^
Moderation components^3^				
Refined grains	10	7.9 (4.8–10)	0.176	0.108^P^
Sodium	10	6.6 (0.8–10)	-0.013	0.908^P^
Added sugar	10	5.5 (1.4–7.6)	0.168	0.123^P^
Saturated fat	10	0.1 (0–3.6)	0.033	0.764^P^

^1^ Variable presented as mean ± SD or median (25^th^-75^th^ percentile).

^2^ Group of foods for which intake is encouraged.

^3^ Group of foods for which intake must be limited.

HEI: Healthy Eating Index.

^P^ Pearson correlation coefficient.

* Statistically significant (p<0.05).

### Bivariate and multivariate analysis

The bivariate analysis between participants’ general characteristics and serum adiponectin in **Table 1** indicates that a history of exclusive breastfeeding, BMI for age and sex percentile category, and abdominal obesity status variables could become confounding factors of serum adiponectin (p-value < 0.25). We calculated the model’s power with an effect size of 0.15, an alpha of 0.05, and four predictors [34] before performing multivariate analysis. The result showed that we needed at least 85 subjects to achieve a power 0f 0.80; hence, we can proceed with the multivariate analysis. Subsequently, in the multiple linear regression analysis, we found that HEI 2015 score was significantly associated with serum adiponectin either before or after adjusted with confounders (β = 0.114; 95% CI = 0.01–0.22; p = 0.037; adjusted β = 0.115; 95% CI = 0.01–0.22; p = 0.032), respectively. A one-unit increase of the HEI 2015 score is associated with a 0.115 μg/ mL increase in serum adiponectin concentration. **(Table 3)** The effect size of this study is 0.094, and the post hoc power calculation is 70%.

**Table 3:** Multiple linear regression analysis between Healthy Eating Index 2015 and serum adiponectin (n = 85).

Model^¶^	Variable	Serum adiponectin (n = 85)		
		β	95% CI	P
	HEI 2015 score (*unadjusted*)	0.114	0.01–0.22	0.037*
**1**	HEI 2015 score (*adjusted*)	0.094	-0.01–0.20	0.087
	History of exclusive breastfeeding^1^	-1.695	-3.44–0.04	0.056
	BMI for age and sex percentile category^2^	-0.413	-2.11–1.29	0.630
	Abdominal obesity status^3^	-1.502	-4.07–1.07	0.248
**2**	HEI 2015 score (*adjusted*)	0.096	-0.01–0.20	0.080
	History of exclusive breastfeeding^1^	-1.687	-3.42–0.04	0.056
	Abdominal obesity status^3^	-1.754	-4.09–0.58	0.139
**3**	HEI 2015 score (*adjusted*)	0.115	0.01–0.22	0.032*
	History of exclusive breastfeeding^1^	-1.547	-3.28–0.19	0.080

^¶^ Model 1: HEI 2015 score was adjusted with history of exclusive breastfeeding, BMI for age and sex percentile category, and abdominal obesity status; Model 2: HEI 2015 score was adjusted with history of exclusive breastfeeding and abdominal obesity status; Model 3: HEI 2015 score was adjusted with history of exclusive breastfeeding.

^1^ History of exclusive breastfeeding: (1) < Six months, (2) ≥ Six months.

^2^ Body Mass Index for age and sex percentile category: (1) <25^th^ percentile = underweight, (2) 25^th^-85^th^ percentile = normal weight, (3) >85^th^ percentile = risk of overweight to overweight.

^3^ Abdominal obesity status based on waist circumference reference for age and sex: (1) No (<80^th^ percentile), (2) Yes (≥80^th^ percentile).

* statistically significant (p<0.05).

The bivariate analysis between serum adiponectin and HEI 2015 score was presented in **Table 2**. The relationship between some of the HEI 2015 components, specifically total fruit, whole fruit, total vegetables, refined grains, and added sugar component with the adiponectin level, had p-value < 0.25 (**Table 2**), thus met the criteria for further assessment by multivariate analysis. As there was collinearity between total fruit and whole fruit components, we only included the whole fruit component in the multivariate analysis. We obtained a significant association between refined grain and added sugar component score with serum adiponectin level from the multivariate analysis (**Table 4**).

**Table 4:** Multiple linear regression analysis between the component score of Healthy Eating Index 2015 and serum adiponectin (n = 85).

Model^¶^	Variable	Serum adiponectin (n = 85)		
		Β	95% CI	P
**1**	Whole fruit	0.468	-0.08–1.02	0.096
	Total vegetable	-0.519	-1.28–0.24	0.180
	Refined grains	0.248	-0.04–0.53	0.086
	Added sugar	0.345	0.07–0.62	0.014*
**2**	Whole fruit	0.466	-0.09–1.02	0.099
	Refined grains	0.293	0.02–0.57	0.039*
	Added sugar	0.316	0.05–0.58	0.022*
**3**	Refined grain	0.316	0.04–0.59	0.027*
	Added sugar	0.302	0.03–0.57	0.030*

^¶^ Model 1: Adiponectin level was predicted by whole fruit, total vegetable, refined grains, and added sugar score variable; Model 2: Adiponectin level was predicted by whole fruit, refined grains, and added sugar score variable; Model 3: Adiponectin level was predicted by refined grains and added sugar score variable.

* statistically significant (p<0.05).

## Discussion

In the present study, the dietary quality of preschool children aged three to five years according to the HEI 2015 score was positively associated with serum adiponectin level both before and after adjustments were made for exclusive breastfeeding history. A one-point increase in the HEI 2015 score was associated with a 0.115 μg/mL increase in serum adiponectin concentration. This association occurred in children with a low average of dietary quality (33.2 ± 8.3 points) and within a normal range of adiponectin level (10.3 ± 4.1 μg/mL).

Our study utilized the 2015 version of HEI, which is a measure of diet quality used to assess how a set of food aligns with a key recommendation of the 2015 to 2020 Dietary Guidelines for Americans. Even though the HEI has not been validated in Indonesia, the assessment had previously been deployed to assess Indonesian women of reproductive age by Stefani et al. [25], who compared American and Indonesian dietary guidelines and found a similarity in some of the food items in both guidelines (i.e., portions of staple food, total protein, vegetables, fruits, and oils). In Indonesia, children older than two years old usually consume a similar type of adult food, albeit in a smaller portion size. Therefore, the use of this index among Indonesian children is appropriate.

The poor HEI score recorded in our current study are consistent with findings of our previous studies on dietary quality in the Indonesian population, such as those in adolescent girls in West Java reported by Agustina et al. [51], and reproductive-aged women in West Sumatera and West Java reported by Stefani et al. [25]; and mothers and children in rural Komodo District, Eastern Indonesia reported by Gibson et al. [52]. These studies also demonstrated that the Indonesian diet quality is still largely inadequate and requires improvement. Stefani et al. examined the HEI of Indonesian women in two regions with different predominant diets and found that the total HEI score was 32.9 ± 7.4 points [25]. Notably, this study [25] and ours recorded similar total HEI score results. Moreover, we noticed that total fruit, whole fruit, whole grains, and food rich in unsaturated fatty acids were the components that garnered the lowest score. This indicates that dietary quality issues are similar in adults or children, although there is no published study using HEI gathered among children in Indonesia to compare with ours. However, other studies in LMICs have reported similar findings [53–55].

Until now, serum adiponectin’s reference values for the pediatric population in Indonesia were not available. Therefore, we used adiponectin threshold values from the IDEFICS cohort study involving European children for our assessment [46] and found that the mean serum adiponectin levels of our participants were within the reference range. Some children in our study exhibited a low level of adiponectin, which positively correlated with low HEI scores. Previous studies have reported that overweight children with lower adiponectin levels may be at higher risk for metabolic diseases in the future [56]; this risk is more prominent in children who are already overweight or obese than in normal-weight children [56–58].

To our knowledge, studies that have evaluated the relationship between HEI and adiponectin levels, especially in children, are not yet available. Guillermo et al. found that higher diet quality scores were associated with 6% to 16% higher adiponectin levels in a multiethnic adult and elderly population in Hawaii and California (p < 0.01) [59]. Volp et al. also observed a significant association between adiponectin concentration and HEI 2010 scores in a healthy young adult population in Brazil (p = 0.02) [17]. Even though both studies were performed in the adult population, the results align with ours.

In our study, a one-point increase in HEI score was only associated with a 0.115 μg/ml increase in adiponectin. However, this little increment can be clinically relevant when it relates to the risk of NCDs onset. As seen in our study, the minimum HEI target score was 80 points, while the mean HEI score was around 33 points; an increase in the HEI score of much as 47 points can be associated with roughly a 5-μg/ml increase in adiponectin level. Kim et al. [60] and Song et al. [61] discovered that increases in plasma adiponectin of much as 1.0 μg/mL and 4.5 μg/ml were associated with a 6% reduction in hypertension prevalence and 38% to 63% lower odds of incident and persistent metabolic syndrome in the adult population, respectively. Similar detailed studies in the children population, however, are still rare, although one of interest is a longitudinal study by Hooshmand et al. [62], who found that the incidence of metabolic syndrome in children in the highest quartile was lower than that in the lowest quartile for modified HEI score (odds ratio: 0.35, 95% confidence interval: 0.13–0.98, p for trend = 0.025) [62]. However, more studies on the pediatrics’ adiponectin biological plausibility are needed to enrich the evidence.

The associations between HEI score and adiponectin level are indirectly influenced by each HEI component’s biological relationship with adiponectin. In the sub-analysis, we found that refined grain and added sugar components can predict the adiponectin value by 6.3%. A one-unit increase in the refined grain and added sugar component scores was associated with an increase of 0.316 μg/mL and 0.302 μg/mL, respectively, in the serum adiponectin concentration. These results indicate that adiponectin level will be higher if the intake of added sugar or refined grains is less, which is consistent with the results of previous studies [17, 63, 64]. These findings confirmed the necessity of maintaining a healthy diet for the regulation of adiponectin.

Our study found that the consumption of fiber-rich food like fruits, vegetables, and whole grains by children was low. This finding was in line with that of a previous study assessing the Indonesian population’s intake, where the fruits and vegetables consumption were inadequate according to Indonesia’s recommendations [65]. Fiber-rich food intake is related to adiponectin due to an enzyme cofactor that mediates oxidative and inflammatory responses in the endothelium. Fiber also changes the intraluminal bacterial balance that further causes a reduction of inflammatory cytokines [66]. Meanwhile, the inverse association between added sugar intake and adiponectin is probably related to adipose tissue accumulation, especially in the intrabdominal area [67]. Cytokines derived from adipose tissue are expected to reduce adiponectin levels, thus inducing inflammation and insulin resistance, which are the underlying causes of metabolic problems [66].

Our findings suggest that diet quality indicators can be useful tools for predicting the risk of NCDs. This study also shows that designing interventions to improve diet quality can be beneficial even when introduced at an early age. To date, specific dietary guidelines for children in Indonesia are not yet available. Therefore, our findings might serve as a preliminary reference for public policymakers seeking to develop relevant and applicable pediatric nutrition guidelines to improve all Indonesian children’s dietary quality. Because HEI provides a good indication for adiponectin concentration, it will become essential for the pediatric or other health practitioners to pay attention to children’s diet quality since their awareness in using a dietary index to assess children’s diet quality is still low. An education program tailored according to our findings, involving parents, teachers, schools, and local health centers, may also help to improve the quality of children’s dietary intake and increase adherence to dietary guidelines from an early age.

Our study’s largest potential limitation was the unavailability of a complete and validated food database that could cover all participants’ foods. To overcome this issue, we created a food database based on the type of meals, snacks, and beverages consumed by our participants obtained during the 24-hour food recall assessment. As a result, we were able to minimize bias during food intake analysis. Meanwhile, another issue that arose was whether the small sample size could capture the same condition in a broader preschool-age children population. Despite the significant association, this study’s effect size was small (0.094), with a post-hoc power calculation of 70%. Although more extensive studies will be needed, our study findings can still suggest the diet and adiponectin profile of the broader population of children in Indonesia and other LMICs. Across Indonesia, diets are shifting towards containing higher energy-dense foods, fats, animal products, and processed foods [68] and this is also seen in LMICs [54]. Food accessibility, food affordability, and convenience factors may bridge the food supply’s influence on people’s diet quality and influence their eating habits preference [69]. Our study observed that caregivers tended to buy ready-to-eat food rather than providing home-cooked food and found that it is easier for young children to obtain unhealthy snacks outside the home. These findings were in line with those other studies involving urban communities [70, 71]. Notably, our study results from Jakarta’s urban megapolitan city are more representative for describing the urban community’s unique risk factors, which are different from those of rural communities. Further assessments of rural communities are still necessary to generalize our study results to all Indonesian children.

Finally, future studies need to include a broader age range of children with different nutritional statuses and socioeconomic backgrounds. This way, the data collected can be more comprehensive in capturing all Indonesian children’s dietary intake and adiponectin profile. We also recommend that dietary intervention research seek to drive the delivery of nutrition education to parents, the training of primary health care providers, and the introduction of healthy lunchbox programs in school. Multicomponent interventions based on our study findings to better confirm the relationship between diet and adiponectin may also be warranted.

## Conclusion

In conclusion, better adherence of young children to a healthy diet has a positive association with their adiponectin level. This result suggests that strengthening children’s dietary quality from an early age by involving all parties in the children’s environment (e.g., parents, teachers at school, policymakers) may help to reduce the risk of NCDs later on in childhood and during adult life.

## Supporting information

S1 Table Sample representativeness analysis. (DOCX)

S1 Data Dietary quality and adiponectin in children. (XLSX)
