# Supplementary material for: Dietary quality score is positively associated with serum adiponectin level in Indonesian preschool-age children living in the urban area of Jakarta
Source: PLoS One. 2021 Feb 4;16(2):e0246234. doi: 10.1371/journal.pone.0246234 (PMC7861444; doi:10.1371/journal.pone.0246234)
Supplement: S1 Table — (DOCX) [file pone.0246234.s001.docx]

Table of Sample Representativeness

|  | I | | | II | | | III | | |
| --- | --- | --- | --- | --- | --- | --- | --- | --- | --- |
| Variables | Initial sample of the current study (n=127)  n (%) | East Jakarta population  % | p-value^*^ | Responder  (n=85)  n (%) | Initial sample of the current study (n=127)  n (%) | p-value^*^ | Non-responder  (n=40)  n (%) | Responder (n=85)  n (%) | p-value^*^ |
| Gender |  |  | 0.887 |  |  | 0.587 |  |  | 0.332 |
| Female | 61 (48) | 49 |  | 44 (51.8) | 61 (48) |  | 17 (42.5) | 44 (51.8) |  |
| Male | 66 (52) | 51 |  | 41 (48.2) | 66 (52) |  | 23 (57.5) | 41 (48.2) |  |
| Mother's education level^1^ |  |  | 0.500 |  |  | 0.889 |  |  | 0.627 |
| Low | 40 (31.5) | 36 |  | 26 (30.6) | 40 (31.5) |  | 26 (30.6) | 14 (35) |  |
| Medium to high | 87 (68.5) | 64 |  | 59 (69.4) | 87 (68.5) |  | 59 (69.4) | 26 (65) |  |
| Father's education level^1^ |  |  | 0.912 |  |  | 0.700 |  |  | 0.443 |
| Low | 36 (28.3) | 29 |  | 22 (25.9) | 36 (28.3) |  | 13 (32.5) | 22 (25.9) |  |
| Medium to high | 91 (71.7) | 71 |  | 63 (74.1) | 91 (71.7) |  | 27 (67.5) | 63 (74.1) |  |
| Nutritional status |  |  | 0.475 |  |  | 0.713 |  |  | 0.463 |
| Under to normal weight | 114 (89.8) | 92.66 |  | 75 (88.2) | 114 (89.8) |  | 37 (92.5) | 75 (88.2) |  |
| Overweight to obese | 13 (10.2) | 7.34 |  | 10 (11.8) | 13 (10.2) |  | 3 (7.5) | 10 (11.8) |  |
| Household income level^2^ |  |  |  |  |  | 0.717 |  |  | 0.429 |
| Low, n (%) |  |  |  | 51 (60) | 73 (57.5) |  | 21 (52.5) | 51 (60) |  |
| Sufficient, n (%) |  |  |  | 34 (40) | 54 (42.5) |  | 19 (47.5) | 34 (40) |  |
| ^1^ Low (below or equal to the 9-year compulsory education); Medium to high (over the mandatory 9-year education) | | | | | | | | | |
| ^2^ Low: below the provincial minimum wage of Jakarta 2018 (< IDR 3,648,035 or < USD 250); Sufficient: equal to or over the provincial minimum wage of Jakarta 2018 (≥ IDR 3,648,035 or ≥ USD 250) | | | | | | | | | |
| I: Baseline characteristics comparison between the initial sample of the current study (n=127) and East Jakarta population; II: Baseline characteristics comparison between responder (n=85) and the initial sample of the current study (n=127); III: Baseline characteristics comparison between non-responder (n=40) and responder of the current study (n=85) | | | | | | | | | |
| * Z-test for two proportion | | | | | | | | | |
